# Interaction Between DHCR24 and hsa_circ_0015335 Facilitates Cognitive Impairment in Cerebral Small Vessel Disease Patients

**DOI:** 10.1111/cns.70131

**Published:** 2024-11-22

**Authors:** Yachen Shi, Min Xu, Xiaoxuan Zhang, Yan Han, Guangjun Xi, Haixia Mao, Jingyu Deng, Qianqian Gao, Yi Ji, Xuemei Ma, Mingyu Li, Chao Cheng, Xiangming Fang, Feng Wang

**Affiliations:** ^1^ Department of Neurology, The Affiliated Wuxi People's Hospital of Nanjing Medical University, Wuxi People's Hospital, Wuxi Medical Center Nanjing Medical University Wuxi China; ^2^ Department of Interventional Neurology, The Affiliated Wuxi People's Hospital of Nanjing Medical University, Wuxi People's Hospital, Wuxi Medical Center Nanjing Medical University Wuxi China; ^3^ Department of Radiology, The Affiliated Wuxi People's Hospital of Nanjing Medical University, Wuxi People's Hospital, Wuxi Medical Center Nanjing Medical University Wuxi China; ^4^ Department of Neurosurgery, The Affiliated Wuxi People's Hospital of Nanjing Medical University, Wuxi People's Hospital, Wuxi Medical Center Nanjing Medical University Wuxi China

**Keywords:** brain magnetic resonance imaging, cerebral small vessel disease, cholesterol metabolism, circRNA, cognitive impairment, mRNA

## Abstract

**Aims:**

The study attempted to determine the underlying role and regulation mechanism of 3β‐hydroxysterol‐Δ24 reductase (DHCR24) in the pathophysiology of cerebral small vessel disease‐associated cognitive impairment (CSVD‐CI). An RNA high‐throughput sequencing and independent verification were conducted to identify potential circRNAs becoming the upstream regulator.

**Methods:**

RNA sequencing was performed in whole‐blood samples in cohort 1 (10 CSVD‐CI and 8 CSVD with cognitively normal [CSVD‐CN] patients). The DHCR24 and candidate circRNAs were verified in an independent cohort 2 (45 CSVD‐CI participants and 37 CSVD‐CN ones). The study also analyzed comprehensive cognitive assessments, plasma molecular index, and brain structure imaging.

**Results:**

The expression of DHCR24 and has_circ_0015335 in whole‐blood samples of CSVD‐CI patients was significantly reduced compared to CSVD‐CN patients in RNA sequencing and independent verification. Furthermore, the levels of DHCR24 and has_circ_0015335 were significantly related to global cognitive impairment in CSVD‐CI patients. Meanwhile, DHCR24 could regulate the correlation between has_circ_0015335 expression and alterations in brain cortex in surface area, thickness, and volume in CSVD‐CI patients. Additionally, hsa_circ_0015335 interacted with DHCR24 for plasma 24(S)‐hydroxycholesterol levels among CSVD‐CI patients.

**Conclusion:**

Interaction between DHCR24 and hsa_circ_0015335 cognitively impaired CSVD by affecting brain cholesterol metabolism and brain structural changes.

## Graphical abstract

Hsa_circ_0015335 mediate DHCR24 levels may facilitate cognitive impairment of cerebral small vessel disease through affecting brain cholesterol metabolism and brain structure changes.

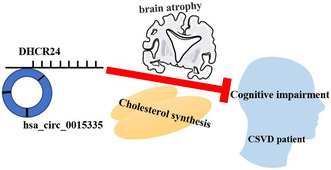

## 1 Introduction

Cerebral small vessel disease (CSVD) is the most common pathology behind vascular cognitive impairment and the usual dementia cause in the elderly [1, 2, 3]. Clinically accepted magnetic resonance imaging (MRI) of CSVD includes lacunar infarcts, white matter hyperintensities (WMH), cerebral microbleeds, enlarged perivascular spaces, and brain atrophy [4]. Among these, WMH is a major risk factor for incident cognitive decline in CSVD [5, 6, 7]. A previous report indicates that the prevalence of imaging features of CSVD with moderate‐to‐severe WMH is 58.4% among dementia subjects [8]. Likewise, CSVD patients with severe WMH are significantly linked with impaired cognition [9]. Despite the public health importance of CSVD‐associated cognitive impairment (CSVD‐CI), only a few proven treatments exist to improve cognitive decline. The limited understanding of the potential pathophysiology of CSVD‐CI remains the primary factor for the lack of effective intervention. However, some underlying pathogenic basis, such as oxidative stress and inflammation, remains concerning [10]. Furthermore, several imaging and blood biomarkers have been identified to evaluate the cognitive decline in CSVD. These include peak width of skeletonised mean diffusivity [11], glial fibrillary acidic protein [12], and neurofilament light chain [13]. However, no sensitive and accurate biomarkers can facilitate the early diagnosis of CSVD‐CI. The management of cognitive impairment by controlling vascular risk factors and using anti‐dementia drugs may be beneficial in the early stages of CSVD‐CI [14]. Therefore, identifying early cognitive impairment is vital in the clinical practice of CSVD.

3β‐hydroxysterol‐Δ24 reductase (DHCR24), encoded by the DHCR24 gene, converts desmosterol to cholesterol and helps maintain cholesterol homeostasis by controlling cholesterol biosynthesis [15, 16]. Reduced DHCR24 expression can cause brain cholesterol deficiency and desmosterol accumulation during brain cholesterol synthetic metabolism [17]. Brain cholesterol deficit can disrupt membrane lipid rafts and intracellular organelles, inducing neuronal pathological impairments and brain dysfunction [18, 19]. Furthermore, the augmentation of DHCR24 levels can provide neuroprotection against the loss of neurons under acute stress conditions [20]. Recent studies demonstrate that DHCR24 knockdown can aggravate tau hyperphosphorylation in the astrocyte [21]. Thus, DHCR24 overexpression can alleviate cholesterol loss, reverse cognitive impairment, and prevent neuronal pathology, including amyloid‐β deposition, synaptic injuries, etc. [22]. Increasing evidence supports that DHCR24 may affect the occurrence and development of cognitive impairment [19]. However, no study illustrates the underlying role of DHCR24 in CSVD‐CI pathophysiology.

DHCR24‐mediated cholesterol biosynthesis is controlled by multiple molecular biological mechanisms, such as phosphorylation and epigenetic factors. DHCR24 can be phosphorylated at multiple sites (T110, Y299, and Y507) to suppress enzyme activity [23]. Additionally, microRNA (miRNA)‐7 can mediate posttranscriptionally the DHCR24 gene while decreasing the DHCR24 expression in the brain [24]. Circular RNA (circRNAs) are closed circular molecules produced from precursor mRNA back‐splicing [25]. CircRNAs can control different biological processes of the body due to their unique covalently closed loop structure, including interacting with miRNA or RNA‐binding proteins while affecting the translation of downstream messenger RNAs (mRNAs) [26]. CircRNAs is a crucial regulator with a pathophysiological role in different neuropsychiatric disorders, e.g., Alzheimer's disease [27] and depression [28]. Previous studies indicate that some circRNAs, e.g., circPTK2 [29] and circEZH2 [30], can regulate the expression of the DHCR24 gene to affect cholesterol synthesis through sponging miRNAs. Hence, the regulated mechanism of DHCR24 expression in CSVD‐CI remains uncertain.

The present study attempted to investigate the possible role and potential upstream regulation of DHCR24 in CSVD‐CI. RNA high‐throughput sequencing was performed between CSVD‐CI and CSVD among cognitively normal (CSVD‐CN) patients to determine differential DHCR24 and potential circRNA expression linked with cognitive decline in CSVD patients. Furthermore, the clinical value of DHCR24 and the regulated molecules of CSVD‐CI were analyzed according to the apparent, microscopic, and mesoscopic characteristics.

## 2 Materials and Methods

### 2.1 Participants

Around 100 CSVD patients of Chinese Han ethnicity were recruited from the Affiliated Wuxi People's Hospital of Nanjing Medical University (Wuxi, China), involving Cohort 1 (18 participants) and Cohort 2 (82 participants). All the participants underwent a standardized clinical interview (such as demographic inventory and physical and mental health examination), brain MRI, neuropsychological assessments, and blood collection.

The inclusion criteria involved: (I) age 50–80 years; (II) education years ≥ 6; and (III) adequate vision and audition for neuropsychological assessments. Based on previously established criteria [31, 32], CSVD patients were determined according to brain MRI changes of white matter hyperintensity (WMH). The Fazekas score is a qualitative tool for estimating WMH severity [33, 34]. Periventricular and deep WMH were quantified with the Fazekas scale (total score is 6), and CSVD patients with total WMH Fazekas scores > 2 were included in the study.

The exclusion criteria were: (I) cerebrovascular disorders with large intracranial vascular lesions or a history of severe cerebrovascular diseases; (II) other neurologic diseases (e.g., Alzheimer's disease, Parkinson's disease) or any brain trauma; (III) any psychiatric disorders (e.g., depressive disorder, schizophrenia), or a family history of psychosis; (IV) severe medical problems (e.g., abnormal liver, kidney, heart, or thyroid function, tumor, immunological disorders); (V) abuse or alcohol or drug dependence; and (VI) any MRI contraindications, e.g., claustrophobia.

The Ethics Committee of the Affiliated Wuxi People's Hospital of Nanjing Medical University approved the current study (approval number: KY2112). All participants or their legal guardians provided written informed consent. All clinical investigations were conducted in strict adherence to the principles outlined in the Declaration of Helsinki, and all experiments were performed in accordance with relevant guidelines and regulations.

### 2.2 Neuropsychological Assessments

Two trained investigators completed the present neuropsychological assessments. The present neuropsychological battery included five parts (11 tests): (I) global cognition (Mini‐Mental State Examination [MMSE] and Montreal Cognitive Assessment [MoCA]); (II) episodic memory (Auditory Verbal Learning Test‐immediate recall [AVLT‐IR] and Auditory Verbal Learning Test‐20‐min delayed recall [AVLT‐20 min DR]); (III) information processing speed (Trail Making Tests A [TMT‐A] and Stroop Color and Word Test A and B [Stroop‐A and Stroop‐B]); (IV) executive function (Trail Making Tests B [TMT‐B], Stroop Color and Word Test C [Stroop‐C], and Digit Span Test [DST]); and (V) visuospatial function Clock Drawing Test [CDT].

Despite the MMSE and MoCA scores, the raw scores of other scales were standardized with Z‐transformed for subsequent analyses. The information processing speed and executive function were quantified using the Z‐transformed scores of relative scales [34, 35, 36]. For example, information processing speed scores were measured using the averages of TMT‐A, Stroop‐A, and Stroop‐B *Z* scores. Moreover, executive function scores were computed with the averages of the TMT‐B, Stroop‐C, and DST‐backward *Z* scores.

Furthermore, the study determined CSVD patients with/without cognitive impairment based on the medical history provided by family members and the outcomes of present neuropsychological assessments. Due to strong evidence supporting MoCA to be superior to MMSE in discriminating between subjects with and without cognitive impairment [37, 38], the MoCA was primarily used for grouping in the present study. According to the recommended criterion of MoCA [39, 40], CSVD patients with a MoCA score < 26 were termed the CSVD‐CI subject. Moreover, those with MoCA scores ≥ 26 were termed the CSVD‐CN subjects. Thus, cohort 1 included 10 CSVD‐CI participants and 8 CSVD‐CN ones, while cohort 2 included 45 CSVD‐CI participants and 37 CSVD‐CN ones.

### 2.3 Collection of Blood Samples

Venous blood was obtained between 8:00 and 9:00 am from each participant after overnight fasting into an EDTA‐coated tube and a PAXgene blood RNA tube (Becton Dickinson).

The blood in an EDTA‐coated tube was centrifuged at 1000 *g* for 10 min at 4°C to obtain plasma within 30 min of collection. Then, the plasma was aliquoted and stored at −80°C. Furthermore, the blood in the PAXgene blood RNA tube was utilized for RNA extraction. The tube was first placed at room temperature for 2 h, then overnight at −20°C, and stored at −80°C until further analysis.

### 2.4 RNA Extraction and RNA High‐Throughput Sequencing

Total RNA was obtained with a PAXgene blood RNA kit (Qiagen) under standardized conditions using a QIAcube (Qiagen) automated processor based on the manufacturer's instructions.

According to the standard Illumina protocols, sequencing libraries and RNA high‐throughput sequencing were generated in all cohort 1 participants (detailed protocol was displayed in Supporting Information). The expression patterns of circRNAs and mRNAs were obtained from the same cohort 1 samples. Subsequent association analyses were conducted and visualized using Cytoscape software (version 3.6.0) [41]. The assembly number of human genome used in the study us hg38.

### 2.5 Analysis of mRNA Qequencing Data

Clean reads were mapped to the reference genome using HISAT2 v2.0.4 software. Mapped reads were assembled using StringTie v1.3.1 software. Assembled transcripts were analyzed for coding potential using the RNA_seQc algorithm. Cuffdiff v2.1.1 software was used to calculate the fragments per kilobase of transcript per million (FPKM) of coding genes in each sample. Gene FPKMs were obtained by summing the FPKMs for transcripts in each gene group. Differentially expressed mRNAs were identified using FPKM values for each coding gene. Differentially expressed mRNAs were identified based on a |log_2_ (fold change)| > 0.58 with an adjusted *p* < 0.05.

### 2.6 Analysis of circRNA Sequencing Data

CircRNAs were identified using CIRI software, followed by matching with data from the circBase database (http://www.circbase.org/). CircRNA expression was normalized using Back Spliced Reads Per million mapped reads, with differential expression analysis accomplished using DEGSEQ software. The differentially expressed circRNAs between the two groups were filtered by |log_2_ (fold change)| > 0.58 and *p* < 0.05 as criteria.

### 2.7 Real‐Time Quantitative Polymerase Chain Reaction (RT‐qPCR)

Primers of mRNA and circRNA have been designed, the details of which are provided in Table S1. The expression levels of mRNA and circRNAs were computed from the threshold cycle (*C*_t_) value. Moreover, the $2^{-\Delta \Delta C_{t}}$ method helped obtain the relative fold change in expression [28]. All the experiments were performed in triplicate. Supporting Informations provides more details.

### 2.8 Enzyme‐Linked Immunosorbent Assay (ELISA) Analyses

The plasma 24(S)‐hydroxycholesterol (24(S)‐OHC) concentration in cohort 2 was measured using a commercial ELISA kit based on the manufacturer's protocols (ADI‐900‐210‐0001; Enzo Biochem Inc. Farmingdale, MY, USA). The concentration in each plate was determined with a microplate reader. The inter‐ and intra‐assay coefficients of variation were < 4%. Each sample had been tested in triplicate.

### 2.9 MRI Data Acquisition, Processing and Analysis

The present study used a 3.0 T MR scanner to acquire a high‐resolution T1‐weighted 3D magnetisation‐prepared rapid gradient echo sequence. Then, MRI data processing was performed using the automated pipeline from FreeSurfer (version 7.3.2) [42, 43]. Cortical surface area, cortical thickness, and gray matter volume in each region of interest of FreeSurfer's atlas were obtained from FreeSurfer's output aparc.stats files. Monte Carlo simulation helped identify the threshold of a given nominal *p*‐value (a cluster size correction of *p* < 0.05). More details as outlined in Supporting Informations.

### 2.10 Statistical Analyses

SPSS version 20.0 software was utilized for all the statistical analyses. The Kolmogorov–Smirnov test helped with normal data distribution. Continuous variables were analyzed using the Mann–Whitney *U* test in cases with a non‐normal distribution. In contrast, the independent samples *t*‐test was used in cases showing a normal distribution. The chi‐square test helped analyze the differences between categorical variables. Pearson correlation analysis helped determine the relationship between the two variables. The predicted value of each participant was measured using binary logistic regression, representing the combined marker according to relative DHCR24 and has_circ_0015335 levels [44]. Meanwhile, linear regression analysis helped evaluate the interaction between relative levels of DHCR24 and has_circ_0015335 on plasma levels of 24(S)‐OHC. Furthermore, mediation analysis in CSVD‐CI patients helped determine whether DHCR24 regulated the association between has_circ_0015335 and changes in brain MRI features according to a standard 3‐variable mediation model [45]. Receiver operating characteristic (ROC) curves were utilized for the diagnostic performance of markers. The Youden index helped estimate the optimal sensitivity and specificity values. The results with *p* < 0.05 (two‐tailed) were statistically significant.

## 3 Results

### 3.1 Clinical Characteristics of Participants

Table 1 demonstrates no difference in age, gender, education years, body mass index, number of smokers, and complications (hypertension and diabetes) between CSVD‐CI and CSVD‐CN patients in cohort 1 and cohort 2. Meanwhile, two CSVD groups in the two cohorts showed no significantly different Fazekas scores.

**TABLE 1:** Clinical characteristics of all participants.

	Cohort 1			Cohort 2		
	CSVD‐CI (*N* = 10)	CSVD‐CN (*N* = 8)	*p*	CSVD‐CI (*N* = 45)	CSVD‐CN (*N* = 37)	*p*
Age	72.1 ± 5.95	73.25 ± 4.06	0.648*	71.18 ± 5.06	69.00 ± 6.53	0.093*
Gender (male/female)	4/6	5/3	0.635^b^	26/19	21/16	0.926^b^
Education (years)	7.60 ± 0.97	8.25 ± 1.58	0.297*	9.07 ± 2.44	10.11 ± 2.39	0.060^a^
Body mass index	23.43 ± 2.87	24.67 ± 4.44	0.223	23.01 ± 2.96	22.59 ± 2.63	0.488*
Hypertension (yes/no)	2/8	1/7	1.000^b^	33/12	24/13	0.407^b^
Diabetes (yes/no)	5/5	4/4	1.000^b^	9/36	6/31	0.318^b^
Smoking (yes/no)	5/5	3/5	0.664	21/24	18/19	0.858^b^
Fazekas score (3/4/5/6)	4/3/2/1	4/1/2/1	0.965^a^	17/18/4/6	23/7/5/2	0.061^a^
MMSE score	24.50 ± 3.27	28.63 ± 1.19	0.004*	25.64 ± 3.29	28.68 ± 1.18	< 0.001^a^
MoCA score	20.40 ± 4.03	28.13 ± 1.46	< 0.001*	20.84 ± 3.86	27.70 ± 1.66	< 0.001^a^
AVLT‐IR (raw score)	3.83 ± 1.39	6.04 ± 1.73	0.008*	3.90 ± 1.31	6.09 ± 1.47	< 0.001*
AVLT‐IR (*Z* score)	−0.52 ± 0.74	0.65 ± 0.92	0.008*	−0.56 ± 0.75	0.68 ± 0.83	< 0.001*
AVLT‐20min DR (raw score)	1.90 ± 1.66	5.25 ± 2.05	0.001*	2.31 ± 2.22	5.59 ± 2.42	< 0.001*
AVLT‐20min DR (*Z* score)	−0.60 ± 0.67	0.75 ± 0.83	0.001*	−0.52 ± 0.78	0.64 ± 0.86	< 0.001*
TMT‐A (raw score)	173.90 ± 119.69	89.25 ± 29.80	0.070*	109.36 ± 46.30	58.92 ± 16.92	< 0.001^a^
TMT‐A (*Z* score)	0.38 ± 1.21	−0.47 ± 0.30	0.070*	0.52 ± 1.05	−0.63 ± 0.39	< 0.001^a^
Stroop‐A (raw score)	48.60 ± 12.45	30.13 ± 7.74	0.004*	42.69 ± 27.67	28.59 ± 6.40	< 0.001^a^
Stroop‐A (*Z* score)	0.59 ± 0.90	−0.73 ± 0.55	0.004*	0.29 ± 1.26	−0.35 ± 0.29	< 0.001^a^
Stroop‐B (raw score)	91.40 ± 35.55	46.88 ± 14.67	0.037*	74.58 ± 37.81	51.16 ± 16.18	< 0.001^a^
Stroop‐B (*Z* score)	0.55 ± 1.00	−0.69 ± 0.41	0.037*	0.33 ± 1.18	−0.40 ± 0.50	< 0.001^a^
Information processing speed	0.51 ± 0.89	−0.63 ± 0.25	0.003*	0.38 ± 1.07	−0.46 ± 0.27	< 0.001^a^
TMT‐B (raw score)	469.00 ± 252.11	198.75 ± 91.13	0.009*	351.62 ± 214.56	151.19 ± 36.14	< 0.001^a^
TMT‐B (*Z* score)	0.51 ± 1.06	−0.47 ± 0.30	0.009*	0.48 ± 1.14	−0.58 ± 0.19	< 0.001^a^
Stroop‐C (raw score)	197.90 ± 119.54	99.13 ± 26.34	0.037*	178.78 ± 98.88	93.43 ± 31.07	< 0.001^a^
Stroop‐C (*Z* score)	0.43 ± 1.17	−0.54 ± 0.26	0.037*	0.44 ± 1.14	−0.54 ± 0.36	< 0.001^a^
DST‐backward (raw score)	3.50 ± 1.51	4.13 ± 0.64	0.292*	3.31 ± 1.18	4.29 ± 0.62	< 0.001^a^
DST‐backward (*Z* score)	−0.23 ± 1.24	0.29 ± 0.53	0.292*	−0.41 ± 1.09	0.50 ± 0.57	< 0.001^a^
Executive function	0.24 ± 0.47	−0.29 ± 0.18	0.007*	0.17 ± 0.60	−0.21 ± 0.22	0.001*
CDT (raw score)	6.30 ± 2.40	8.25 ± 0.89	0.027^a^	8.07 ± 1.59	9.03 ± 1.19	0.001^a^
CDT (*Z* score)	−0.41 ± 1.15	0.52 ± 0.42	0.027^a^	−0.29 ± 1.06	0.35 ± 0.80	0.001^a^
24(S)‐OHC levels (ng/L)	—	—	—	3.78 ± 1.03	3.28 ± 1.00	0.028*

*Note:* Data are presented as the mean ± stand deviation. The information processing speed total scores were calculated by TMT‐A, Stroop‐A, and Stroop‐B scales (*Z* scores), and the executive function total scores were calculated by TMT‐B, Stroop‐C, and DST‐backward scales (*Z* scores).

Abbreviations: 24(S)‐OHC, 24(S)‐hydroxycholesterol; AVLT‐20 min DR, Auditory Verbal Learning Test‐20‐min delayed recall; AVLT‐IR, Auditory Verbal Learning Test‐immediate recall; CDT, Clock Drawing Test; CSVD‐CI, cerebral small vessel disease‐cognitive impairment; CSVD‐CN, cerebral small vessel disease‐cognitively normal; DST, Digit Span Test; MMSE, Mini‐mental State Examination; MoCA, Montreal Cognitive Assessment; Stroop‐A, Stroop Color and Word Test A; Stroop‐B, Stroop Color and Word Test B; Stroop‐C, Stroop Color and Word Test C; TMT‐A, Trail Making Test A; TMT‐B, Trail Making Test B.

* *p*‐values were obtained by Independent‐Sample *T* test.

^a^ *p*‐values were obtained by Mann–Whitney *U* test.

^b^ *p*‐values were obtained by Chi‐square test.

Although the global cognition (MMSE and MoCA) showed significant differences between CSVD‐CI and CSVD‐CN patients in the two cohorts, some cognitive domains depicted slightly different results (Table 1). In cohort 1, except for TMT‐A and DST‐backward, other assessment scores revealed significant differences between CSVD‐CI and CSVD‐CN groups (Table 1). However, in cohort 2, there were significantly elevated TMT‐A/B, Stroop‐A/B/C, executive function, and information processing speed scores. Additionally, there was a significant decline in AVLT‐IR, AVLT‐20 min DR, DST‐backward, and CDT scores in CSVD‐CI patients compared to CSVD‐CN ones (Table 1).

### 3.2 Discovery and Validation of Differentially Expressed mRNA and circRNAs

**FIGURE 1:**
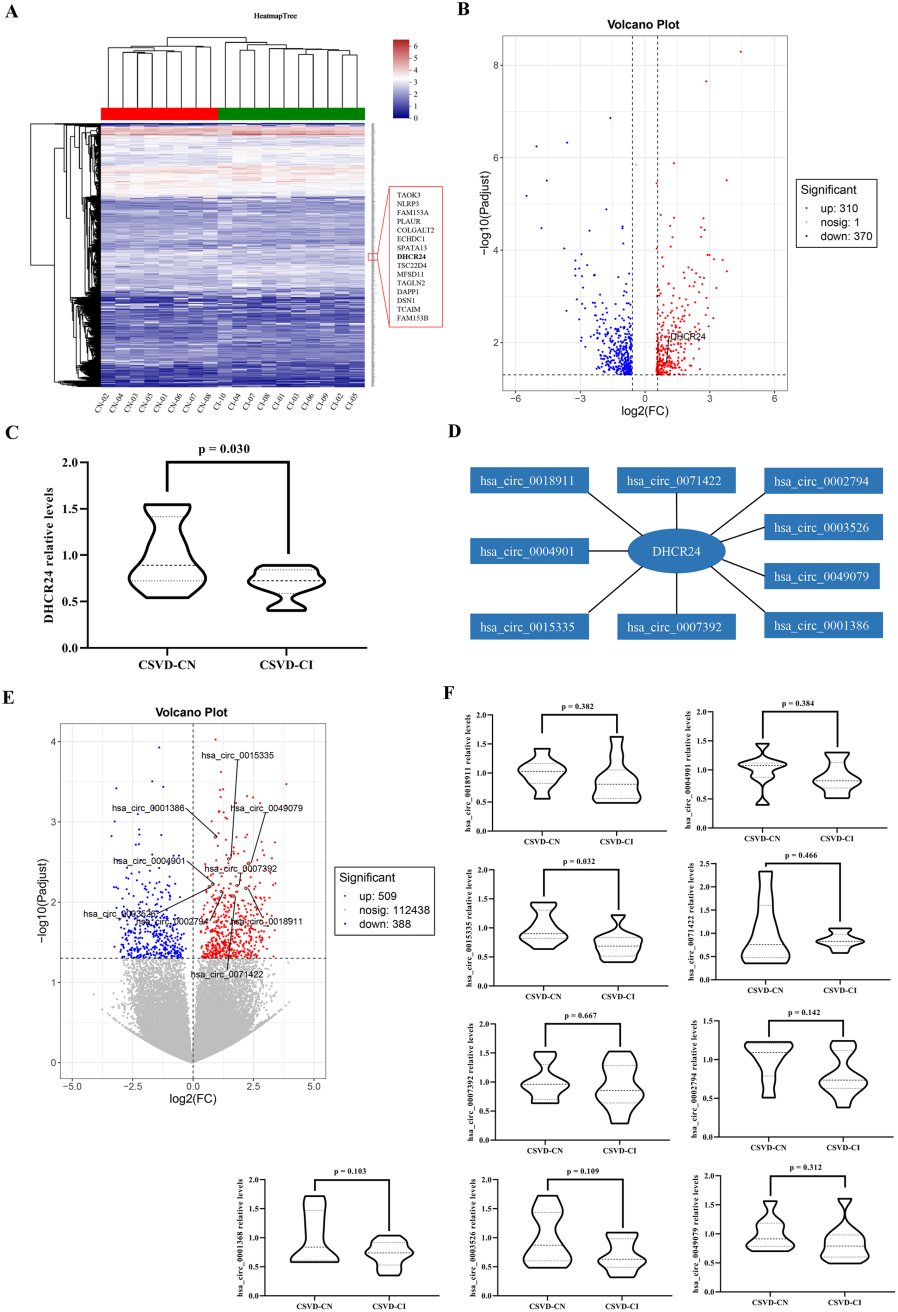
Discovery and validation of differentially expressed DHCR24 and candidate circRNAs in cohort 1. (A) Cluster heat map shows in cohort 1 (8 CSVD‐CN patients and 10 CSVD‐CI patients), 681 differentially expressed mRNAs with a |log_2_ (fold change)| > 0.58 and *p* < 0.05 and an effective test value in any one sample. The bold mRNA is DHCR24. (B) Volcano plot of 681 differentially expressed mRNAs. Horizontal coordinates represent mRNAs with log_2_ (fold change) (CSVD‐CN/CSVD‐CI) > 0.58 or < −0.58. The vertical axis displays −log_10_ (*p*‐value) > 1.30 (mean *p* < 0.05) of differentially expressed mRNAs. Each point represents an individual mRNA. DHCR24 is regulated mRNA in cohort 1. (C) RT‐qPCR was performed to verify the expression of DHCR24 in cohort 1 (8 CSVD‐CN patients and 10 CSVD‐CI patients). Each sample was tested in triplicate. Independent‐sample *t*‐test was used for data analysis and all data represents means ± standard deviation. (D) CircRNA‐associated DHCR24 mRNA networks. (E) Volcano plot of 897 differentially expressed circRNAs. Horizontal coordinates represent circRNAs with |log_2_ (fold change)| > 0.58 (CSVD‐CN/CSVD‐CI). The vertical axis displays −log_10_ (*p*‐value) > 1.30 (mean *p* < 0.05) of differentially expressed circRNAs. Each point represents an individual circRNA. Nine candidate circRNAs were showed in this figure. (F) RT‐qPCR was performed to verify the expression of nine candidate circRNAs in cohort 1 (8 CSVD‐CN patients and 10 CSVD‐CI patients). Each sample was tested in triplicate. Independent‐sample *t*‐test was used for data analysis and all data represents means ± standard deviation. CSVD‐CI, cerebral small vessel disease‐cognitive impairment; CSVD‐CN, cerebral small vessel disease‐cognitively normal; RT‐qPCR, real‐time quantitative polymerase chain reaction.

High‐throughput sequencing identified significantly different 1594 mRNA and 897 circRNAs between CSVD‐CI and CSVD‐CN groups in cohort 1 (SRA accession number: PRJNA831854 or GEO201483; Figure S1). Significantly different 681 mRNAs were screened in cohort 1 using a strict standard to reduce the potential effect of the detection method and identify mRNAs (i.e., each mRNA had an effective test value in a sample) (Figure 1A). DHCR24 was observed in these 681 mRNAs and depicted significantly enhanced expression in CSVD‐CN patients compared to CSVD‐CI patients (Figure 1A,B). Subsequently, DHCR24 expression was validated using RT‐qPCR in original cohort 1 samples. Therefore, the change of DHCR24 relative levels remained consistent with mRNA sequencing (Figure 1C).

According to the differentially expressed circRNAs and mRNAs, circRNA‐associated mRNA networks were developed in cohort 1. Among them, circRNA‐associated DHCR24 networks are depicted in Figure S2 (correlation coefficient > 0.3 and *p* < 0.05). There were 130 candidate circRNAs associated with these networks. This could regulate the underlying function of DHCR24 in vivo. Then, nine candidate circRNAs were screened with complete information from the circBase database of these 130 circRNAs by consulting the circBase database (Figure 1D and Table S2).

In the circRNA sequencing data of cohort 1, nine candidate circRNAs demonstrated significantly elevated expression in CSVD‐CN patients compared to CSVD‐CI patients (Figure 1E). Likewise, these circRNAs could be detected and validated using RT‐qPCR in cohort 1 (Figure 1F). Finally, only one circRNA, i.e., has_circ_0015335, had significantly different expression between CSVD‐CI and CSVD‐CN patients, consistent with the circRNA sequencing results (Figure 1F).

### 3.3 Differentially Expressed DHCR24 and has_circ_0015335 in the Independent Replication Cohort

**FIGURE 2:**
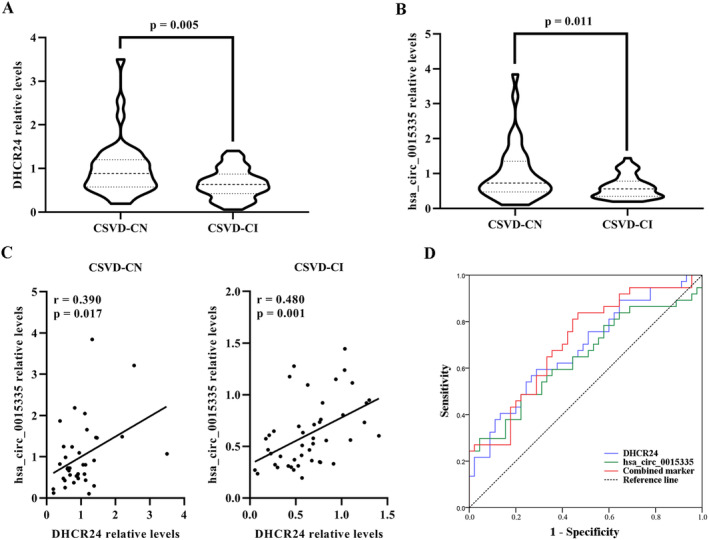
Replication and the clinical application of DHCR24 and has_circ_0015335 in the independent replication cohort 2. (A, B) Expression levels of DHCR24 and has_circ_0015335 between CSVD‐CN and CSVD‐CI groups were determined by RT‐qPCR. (C) Correlations between DHCR24 and has_circ_0015335 expression levels in two groups. (D) ROC curves of DHCR24, has_circ_0015335, and the combination of these two indices. CSVD‐CI, cerebral small vessel disease‐cognitive impairment; CSVD‐CN, cerebral small vessel disease‐cognitively normal; RT‐qPCR, real‐time quantitative polymerase chain reaction; ROC, receiver operating characteristic.

There was a significant increase in the DHCR24 (*p* = 0.005) and has_circ_0015335 (*p* = 0.011) levels in 37 CSVD‐CN patients when compared to 45 CSVD‐CI patients of cohort 2 (Figure 2A,B). These results validated the statistical significance of multiple comparisons (Bonferroni correction *p* < 0.0125). Based on the human reference genome, hsa_circ_0015335 is derived from the exon 14 to exon 17 of the RABGAP1L gene (Figure S3).

Moreover, a positive correlation between DHCR24 and hsa_circ_0015335 levels was observed in 45 CSVD‐CI patients (Figure 2C). However, the DHCR24 levels were not associated with hsa_circ_0015335 levels in CSVD‐CN patients (Figure 2C).

Furthermore, Figure 2D shows the AUC values of DHCR24 and has_circ_0015335 at 0.682 (95% CI: 0.566–0.798) and 0.644 (95% CI: 0.521–0.767). However, combining them could provide a greater diagnostic power with an AUC value of 0.708 (95% CI: 0.595–0.821). This corresponds to a specificity of 53.33% and a sensitivity of 83.78% for MDD diagnosis (Figure 2D).

### 3.4 The Associations of DHCR24 and has_circ_0015335 Expression With Cognitive Assessments

**FIGURE 3:**
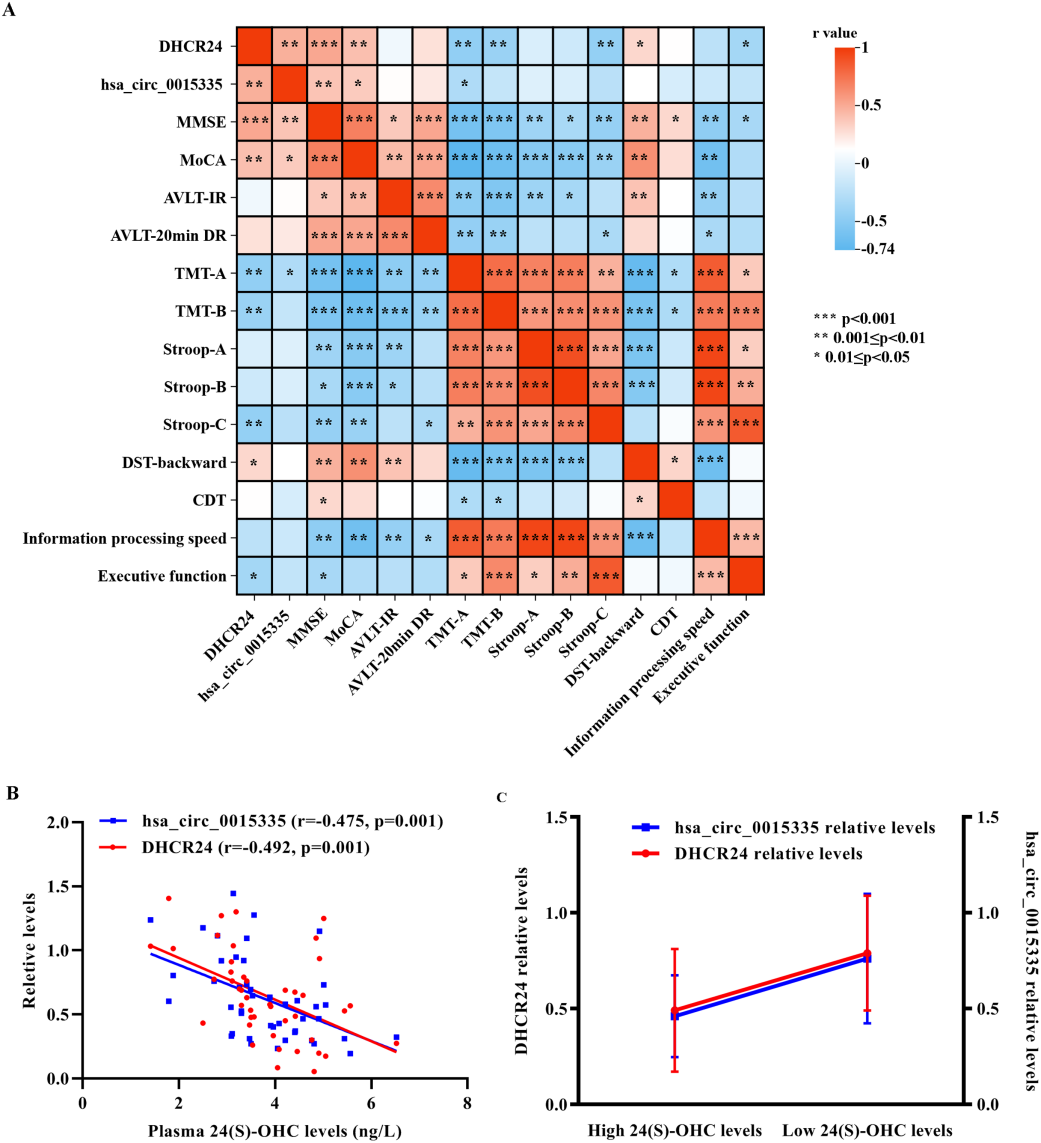
Association analyses of DHCR24 and has_circ_0015335 levels with cognitive assessments and 24(S)‐OHC levels in CSVD‐CI patients. (A) Heatmap of correlation analyses of cognitive assessments with DHCR24 and has_circ_0015335 levels. *0.01 ≤ *p* < 0.05; **0.001 ≤ *p* < 0.01; ***p < 0.001. (B) Correlation analysis of 24(S)‐OHC levels. (C) Interaction analysis of DHCR24 and has_circ_0015335 for the 24(S)‐OHC levels. 24(S)‐OHC, 24(S)‐hydroxycholesterol; AVLT‐20 min DR, auditory verbal learning test‐20‐min delayed recall; AVLT‐IR, auditory verbal learning test‐immediate recall; CDT, Clock Drawing Test; CSVD‐CI, cerebral small vessel disease‐cognitive impairment; CSVD‐CN, cerebral small vessel disease‐cognitively normal; DST, Digit Span Test; MMSE, mini‐mental state examination; MoCA, montreal cognitive assessment; Stroop‐A, Stroop Color and Word Test A; Stroop‐B, Stroop Color and Word Test B; Stroop‐C, Stroop Color and Word Test C; SVD, small‐vessel disease; TMT‐A, Trail Making Test A; TMT‐B, Trail Making Test B.

Correlation analyses depicted that DHCR24 levels were positively associated with MMSE, MoCA, and DST‐backward scores and negatively correlated with TMT‐A/B, Stroop‐C, and executive function scores among 45 CSVD‐CI patients of cohort 2 (Figure 3A). Furthermore, the expression of has_circ_0015335 was negatively correlated with TMT‐A scores while positively correlated with MMSE and MoCA scores (Figure 3A). However, no significant correlation was found between DHCR24 / has_circ_0015335 levels and cognitive assessments in CSVD‐CN patients (data not shown).

### 3.5 Association Analyses of DHCR24 and has_circ_0015335 Levels With Changes in Brain MRI Features

**FIGURE 4:**
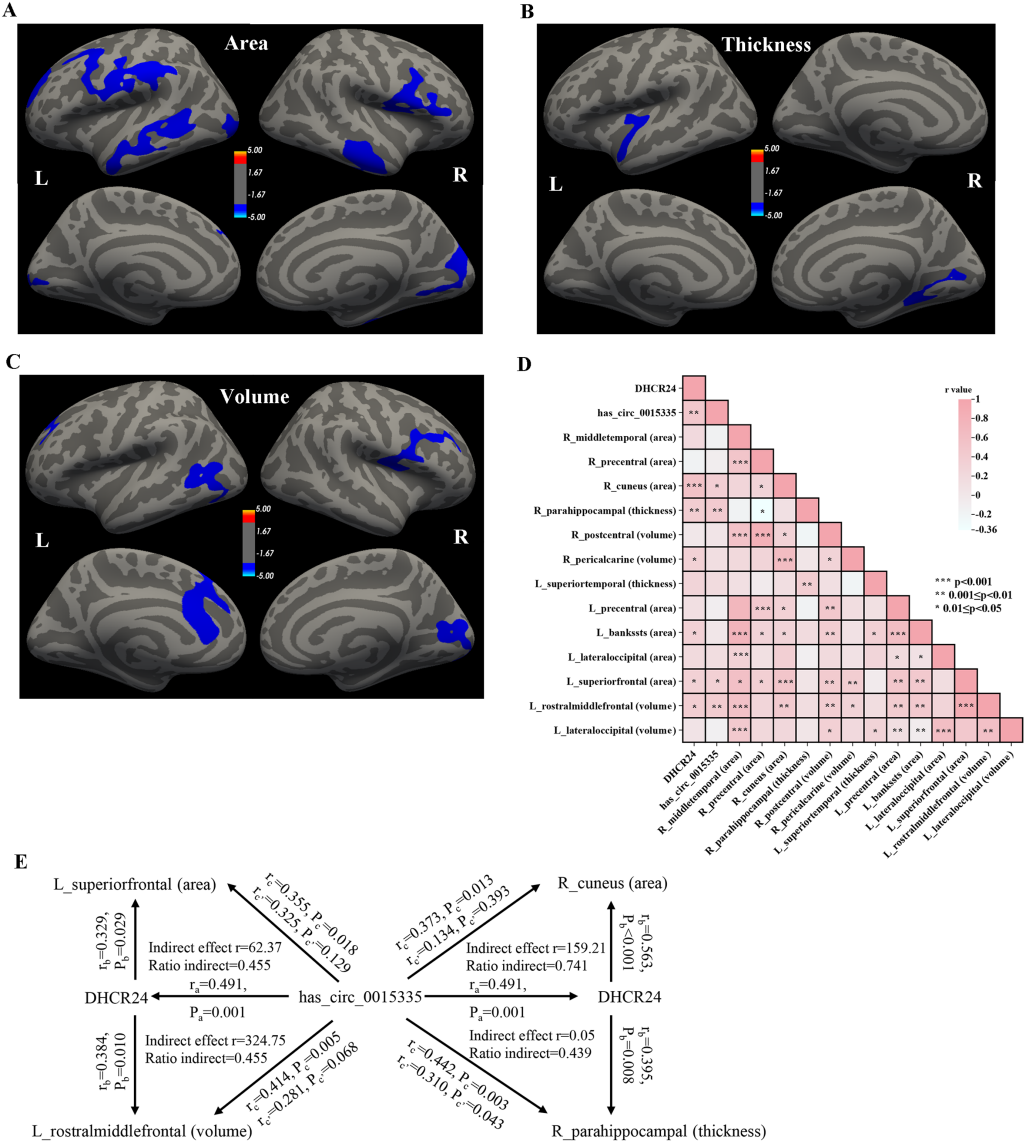
Association analyses of DHCR24 and has_circ_0015335 levels with brain MRI features in CSVD‐CI patients. (A–C) Significant difference of cortical surface area (mm^2^), cortical thickness (mm), and gray matter volume (mm^3^) in brain between CSVD‐CN and CSVD‐CI patients (*p* < 0.05, correction based on Monte Carlo Simulation). (D) Heatmap of correlation analyses of brain MRI features with DHCR24 and has_circ_0015335 levels. *0.01 ≤ *p* < 0.05; **0.001 ≤ *p* < 0.01; ***p < 0.001. (E) The mediation effects of DHCR24 levels on has_circ_0015335 and brain structure changes. “a,” “b,” and “c′” represent the direct effect between the two variables, and “c” represents the total effect between the two variables. CSVD‐CI, cerebral small vessel disease‐cognitive impairment; MRI, magnetic resonance imaging.

Compared with 30 CSVD‐CN patients, 44 CSVD‐CI patients possessed significantly reduced surface area, cortical thickness, and gray matter volume across several brain regions (Figure 4A, corrected *p* < 0.05). More specifically, the right middle temporal, precentral, and cuneus and left precentral, bankssts, lateral occipital, and superior frontal cortex indicated reduced surface area. The right parahippocampal and left superior temporal cortex showed decreasing thickness. Moreover, the right postcentral pericalcarine and left rostral middle frontal and lateral occipital depicted lessened volume.

In the CSVD‐CI group, DHCR24 levels were positively associated with brain cortical surface area (right cuneus, left bankssts, and left superior temporal), thickness (right parahippocampal), and volume (right pericalcarine and left rostral middle frontal) (Figure 4D). Furthermore, significant correlations could be observed between has_circ_0015335 levels and surface area in the right cuneus and left superior temporal, thickness in the right parahippocampal, and volume in the left rostral middle frontal (Figure 4D). However, no significant correlation could be seen between DHCR24 / has_circ_0015335 levels and differential brain MRI features among CSVD‐CN patients (data not shown).

Mediation analyses in CSVD‐CI patients demonstrated that DHCR24 levels significantly mediated has_circ_0015335 levels on the right cuneus and left superior temporal surface area, right parahippocampal thickness, and left rostral middle frontal volume (Figure 4E).

### 3.6 Possible Functional Mechanisms of Action of hsa_circ_0015335 Mediated DHCR24 in CSVD‐CI

Kyoto Encyclopedia of Genes and Genomes functional enrichment analysis was performed according to the differential mRNA sequencing data in cohort 1 (Supporting Informations). Steroid biosynthesis was the significantly enriched functional mechanism observed between CSVD‐CN and CSVD‐CI groups (Figure S4). This indicated that abnormal steroid biosynthesis could be associated with CSVD‐CI occurrence. According to the KEGG database, DHCR24 plays a key role in steroid biosynthesis in organisms (Figure S5) and is associated with cholesterol metabolism.

Due to the circulating 24(S)‐OHC origins in the brain, plasma concentrations of 24(S)‐OHC can become an effective peripheral marker to reflect brain cholesterol metabolism and neuronal degeneration [46, 47, 48]. The plasma 24(S)‐OHC levels were significantly increased in CSVD‐CI patients compared to CSVD‐CN patients in cohort 2 in the present study (Table 1). Additionally, significant correlations were observed between plasma 24(S)‐OHC levels and DHCR24 and has_circ_0015335 levels in CSVD‐CI patients (Figure 3B). Furthermore, linear regression analyses depicted that lower DHCR24 and has_circ_0015335 levels were significantly correlated with the high plasma levels of 24(S)‐OHC in CSVD‐CI patients. Thus, CSVD‐CI patients were divided into low 24(S)‐OHC level (24 patients with a 24(S)‐OHC level < 3.78) and a high 24(S)‐OHC level (21 patients with a 24(S)‐OHC level ≥ 3.78) groups, depicting a specific interactive pattern. CSVD‐CI patients with higher plasma levels among 24(S)‐OHC patients had lower DHCR24 and has_circ_0015335 levels (Figure 3C).

## 4 Discussion

The present study observed that (I) there was significantly reduced expression of DHCR24 and has_circ_0015335 inside the whole‐blood samples of CSVD‐CI patients compared with CSVD‐CN patients; (II) combining DHCR24 with has_circ_0015335 markers provided significant diagnostic power to differentiate CSVD‐CI from CSVD‐CN patients; (III) the DHCR24 and has_circ_0015335 levels significantly correlated with multiple cognitive assessments in CSVD‐CI patients; (IV) the correlation between has_circ_0015335 expression and alterations of brain cortex in surface area, thickness, and volume was mediated by the DHCR24; and (V) the interaction of hsa_circ_0015335 and DHCR24 impacted the steroid metabolism of brain in CSVD‐CI. Hence, the combined effect of DHCR24 and hsa_circ_0015335 could be involved in the potential CSVD‐CI pathophysiology.

The present study determined an underlying interaction of DHCR24 and hsa_circ_0015335 for cognitive impairment among CSVD patients. Particularly, circRNA‐related regulation was novel in CSVD research. Regarding study design, selection of CSVD‐CN and CSVD‐CI cohorts helped investigate internal mechanisms for single exposure (cognitive impairment). Meanwhile, except for clinical manifestation (cognitive assessments), mesoscopic brain imaging and microcosmic molecular changes provided strong evidence to support the potential association of hsa_circ_0015335 with DHCR24 in CSVD‐CI. Furthermore, the reliability of current findings could be improved by RNA high‐throughput sequencing, independent sample verification, and multilevel statistical analyses.

The present study showed significantly increased DHCR24 levels in CSVD‐CN patients compared to CSVD‐CI patients in high‐throughput sequencing and independent sample verification. Therefore, the study was the first to determine the clinically characteristic alterations of DHCR24 expression in cognitive impairment. Consistent with previous animal studies, DHCR24 could be considered a protective factor to block cognitive decline [19, 49]. Meanwhile, CSVD‐CI patients with lower DHCR24 levels displayed worse cognition (such as global cognition, executive function, and partial information processing speed) and reduced surface area, thickness, and volume of some brain cortex. This supports the protective effect of DHCR24 on cognitive decline. Furthermore, DHCR24 could regulate generalized cholesterol synthesis, including in the brain, based on previous studies and our bioinformatics analysis [50, 51]. Similar to a previous study [52], plasma 24(S)‐OHC levels were also significantly higher in cognitively impaired subjects than normal cognition ones in CSVD patients. This could reflect a deficiency in the blood–brain barrier or neuronal degradation‐induced brain cholesterol turnover increase in CSVD‐CI. Moreover, a significant correlation between DHCR24 and 24(S)‐OHC levels could be observed, further supporting that abnormal expression of DHCR24 could affect the brain cholesterol metabolism to induce CSVD‐CI.

The current study observed nine candidate circRNAs involved in circRNA‐associated DHCR24 networks within RNA high‐throughput sequencing of cohort 1. However, only one significantly different circRNA (hsa_circ_0015335) remained after secondary qRT‐PCR detection in the original samples. Through the independent verification in cohort 2, hsa_circ_0015335 showed a significant increase in CSVD‐CN patients compared to CSVD‐CI patients. Further association analyses depicted that lower hsa_circ_0015335 levels were significantly associated with poor global cognition and partial information processing speed. Meanwhile, there were positive correlations between hsa_circ_0015335 levels and brain structure changes associated with cognitive impairment in CSVD‐CI patients [53, 54, 55]. These findings demonstrated that hsa_circ_0015335 could be involved in the occurrence of cognitive characteristics of CSVD‐CI. However, the precise mechanism of hsa_circ_0015335 in CSVD‐CI remains uncertain.

Other than the evidence of bioinformatic analysis, the present study provided multifaceted evidence to determine the underlying association between DHCR24 and hsa_circ_0015335 in CSVD‐CI. Linear regression analysis revealed an interaction between DHCR24 and hsa_circ_0015335 on the 24(S)‐OHC levels. Therefore, the interaction between hsa_circ_0015335 and DHCR24 might implicate the regulation of brain cholesterol metabolism in CSVD‐CI patients. Additionally, in CSVD‐CI patients, DHCR24 mediated the association of hsa_circ_0015335 with multiple brain structure alterations. Thus, DHCR24 is a pivotal factor influencing hsa_circ_0015335 on cognitive impairment in CSVD patients.

DHCR24 and hsa_circ_0015335 are peripheral biomarkers with clinical value for diagnosing CSVD‐CI. Each one showed good diagnostic performance. However, a panel is recommended for optimal differential diagnosis of CSVD‐CI from CSVD‐CN. The combined marker could provide better accuracy and be a potential pre‐screening marker for identifying CSVD patients with a risk of cognitive decline.

The study has several limitations. (I) Possible regulation mechanisms for DHCR24 and hsa_circ_0015335 were supposed to use bioinformatics and clinical data. Thus, further animal and cell research should be performed for functional verification on the regulatory mechanism of these two molecules in subsequent studies. (II) Longitudinal results are not included in the current study because our 3‐year follow‐up is unfinished. Meanwhile, multicenter verification is helpful to provide stronger evidence to support the present findings, which will be conduct in our following study. CSVD patients transiting from noncognitive to cognitive impairment will be concerned with investigating the dynamic change of DHCR24 and hsa_circ_0015335 levels.

## 5 Conclusions

The current study demonstrated that DHCR24 and hsa_circ_0015335 levels were significantly correlated with cognitive impairment in CSVD patients. Based on the clinical, molecular, and imaging evidence, the interaction between DHCR24 and hsa_circ_0015335 may affect the brain cholesterol metabolism and brain structural changes in CSVD‐CI patients.

## Conflicts of Interest

The authors declare no conflicts of interest.

## Supporting information


Data S1.

## Data Availability

The data that support the findings of this study are available from the corresponding author upon reasonable request.
