# Supplementary material for: Interaction Between DHCR24 and hsa_circ_0015335 Facilitates Cognitive Impairment in Cerebral Small Vessel Disease Patients
Source: CNS Neurosci Ther. 2024 Nov 22;30(11):e70131. doi: 10.1111/cns.70131 (PMC11584349; doi:10.1111/cns.70131)
Supplement: Supplementary file 1 — Data S1. [file CNS-30-e70131-s001.docx]

# RNA high-throughput sequencing

## Library preparation and RNA sequencing

Total RNA quality was assessed using agarose gel electrophoresis and quantified using a NanoDrop spectrophotometer (NanoDrop, USA).

Total RNA was processed using a Ribo-ZeroTM Magnetic kit (Epicentre Technologies, Madison, WI, USA) to remove ribosomal RNA (rRNA). The remaining RNA molecules were then processed using a TruSeq RNA Library Preparation kit (Illumina, San Diego, CA, USA) in accordance with the Illumina protocol. Subsequently, real-time polymerase chain reaction (RT-PCR) was performed using Phusion High-Fidelity DNA polymerase, Index (X) Primer and Universal PCR primers. The products were purified using an AMPure XP system. Finally, the quality of the library was evaluated using an Agilent 2100 Bioanalyzer (Agilent Technologies, Palo Alto, CA, USA), and RNA libraries sequenced with 150 bp paired-end read lengths on an Illumina HiSeq 4000 platform (Illumina, San Diego, CA, USA).

Sequence quality was assessed using FastQC software v0.10.1 (http://www.bioinformatics.babraham.ac.uk/projects/fastqc/). Based on the distribution of the low-quality fraction of the Illumina system data, Trim Galore (http://www.bioinformatics.babraham.ac.uk/projects/trim_galore/) was used to trim low-quality bases and adaptors. The expression patterns of circRNAs and mRNAs were analyzed in the present study.

# Real-time quantitative polymerase chain reaction (RT-qPCR)

Specific primers of mRNA and circRNA were designed and synthesized by Shanghai Genesky Biotechnology Company (Supplementary Table 1). cDNA was synthesized using a HiScript Q RT SuperMix for qPCR kit (Vazyme, R123-01), subsequently analyzed by RT-qPCR using a SYBR Green Real-time PCR Master Mix (Roche, Mannheim, Germany). All reactions were performed in triplicate. The expression levels of mRNA and circRNAs were calculated from the threshold cycle (Ct) value, and the relative fold change in expression (CSVD-CI *vs.* CSVD-CN) obtained by the 2^−ΔΔCt^ method. Glyceraldehyde-3-phosphate dehydrogenase was utilized as the endogenous control. Each sample was tested in triplicate.

# Functional Enrichment Analysis

KEGG (Kyoto Encyclopedia of Genes and Genomes) is a knowledge base for systematic analysis of gene functions, linking genomic information with higher order functional information. Functional enrichment analysis was performed using the clusterProfiler package in R software (http://bioconductor.org/packages/release/bioc/html/clusterProfiler.html). The KEGG pathways with p-values of < 0.05 were regarded as significant.

# MRI data acquisition, processing and analysis

## Image acquisition

A 3.0T MR scanner (MAGNETOM Prisma, Siemens Healthcare, Germany) is used in the present study. The data of high-resolution T1-weighted images (T1WI) was analyzed for several brain MRI features. None of subjects had excessive motion artifacts (≥ 2 mm translational or ≥ 2° rotational movements) or incomplete image coverage.

The scanning parameters were as follows: TR = 3000 ms, TE = 2.56 ms, TI = 1100 ms, flip angle = 7°; matrix = 320 × 320; slice number = 208, thickness (gap) = 0.8 mm FOV = 256mm × 256mm.

# MRI processing and analysis

Freesurfer 7.3.2 [1] is an established computational framework in the neuroscience community [2]. This framework contains a routine called recon-all which implements all the required steps to pre-process T1-weighted MR images and extract cortical thickness estimations. In this work, cortical surface reconstructions were computed from these images using the recon-all routine of the FreeSurfer version 7.3.2 (<http://www.surfer.nmr.mgh.harvard.edu/>).

The technical details of these procedures have been described in previous articles [3, 4]. In brief, the first stage is to extract the cortical surface, including removal of non-brain tissue using a hybrid watershed/surface deformation procedure, automated Talairach transformation, segmentation of grey/white matter, and intensity normalisation. The second processing is to model the cortical surface. Segmented white matter volume is used to derive a tessellated surface representing the grey/white matter boundary (inner surface), which is automatically corrected for topology defects and expanded to model the pial–grey boundary (outer surface). After the cortical models are completed, cortical surface area, cortical thickness, and grey matter volume can be measured [5]. Surface measures were smoothed using a Gaussian filter with a 10-mm FWHM.

## Statistical analysis

Cortical surfaces, cortical thickness, and grey matter volume in each region of interest of FreeSurfer’s atlas were obtained directly from FreeSurfer's output aparc.stats files.

T-test was used to compare the differences in cortical surfaces, cortical thickness, and grey matter volume of group changes between CSVD-CN and CSVD-CI groups.

Correction for multiple comparisons was performed through FreeSurfer's mri_glmfit-sim, which conducts the Monte Carlo Simulation to identify the cluster size threshold at a given nominal p value. Clusters were defined by a cluster size correction of p < 0.05. The significant clusters are showed in blue on the whole brain maps (Figure 4A-C).

**Supplementary Table 1. The information of PCR primers.**

| List of oligonucleotide sequences | 5' > 3' |
| --- | --- |
| GAPDH -F | GGAGTCCACTGGCGTCTTCA |
| GAPDH -R | GCAGAGGGGGCAGAGATGAT |
| DHCR24 -F | CACAAGTACGGCCTGTTCCA |
| DHCR24 -R | GCTGCTGGAGTAACCGCTAT |
| hsa_circ_0015335 -F | GACTTACTGCTTTGTGAGGACTCA |
| hsa_circ_0015335 -R | AGATCTTATAGAGCGATTCTTGACC |

**Supplementary Table 2.** **Candidate circRNAs in the sequencing data of the cohort 1.**

| CircRNA_ID | P-value | log_2_FoldChange | Regulation | Type | Gene symbol |
| --- | --- | --- | --- | --- | --- |
| hsa_circ_0015335 | 0.0029 | 1.5637 | Up | exon | RABGAP1L |
| hsa_circ_0001386 | 0.0015 | 1.0118 | Up | exon | CTBP1 |
| hsa_circ_0004901 | 0.0060 | 0.8964 | Up | exon | APAF1 |
| hsa_circ_0003526 | 0.0064 | 0.7601 | Up | exon | SPG21 |
| hsa_circ_0002794 | 0.0074 | 1.2571 | Up | exon | FAM120A |
| hsa_circ_0049079 | 0.0033 | 2.3956 | Up | exon | MYO1F |
| hsa_circ_0007392 | 0.0062 | 1.9054 | Up | exon | DCAF5 |
| hsa_circ_0018911 | 0.0067 | 2.2723 | Up | exon | VDAC2 |
| hsa_circ_0071422 | 0.0084 | 1.7561 | Up | exon | SH3RF1 |

P-value and fold change were calculated by CSVD-CN/CSVD-CI.


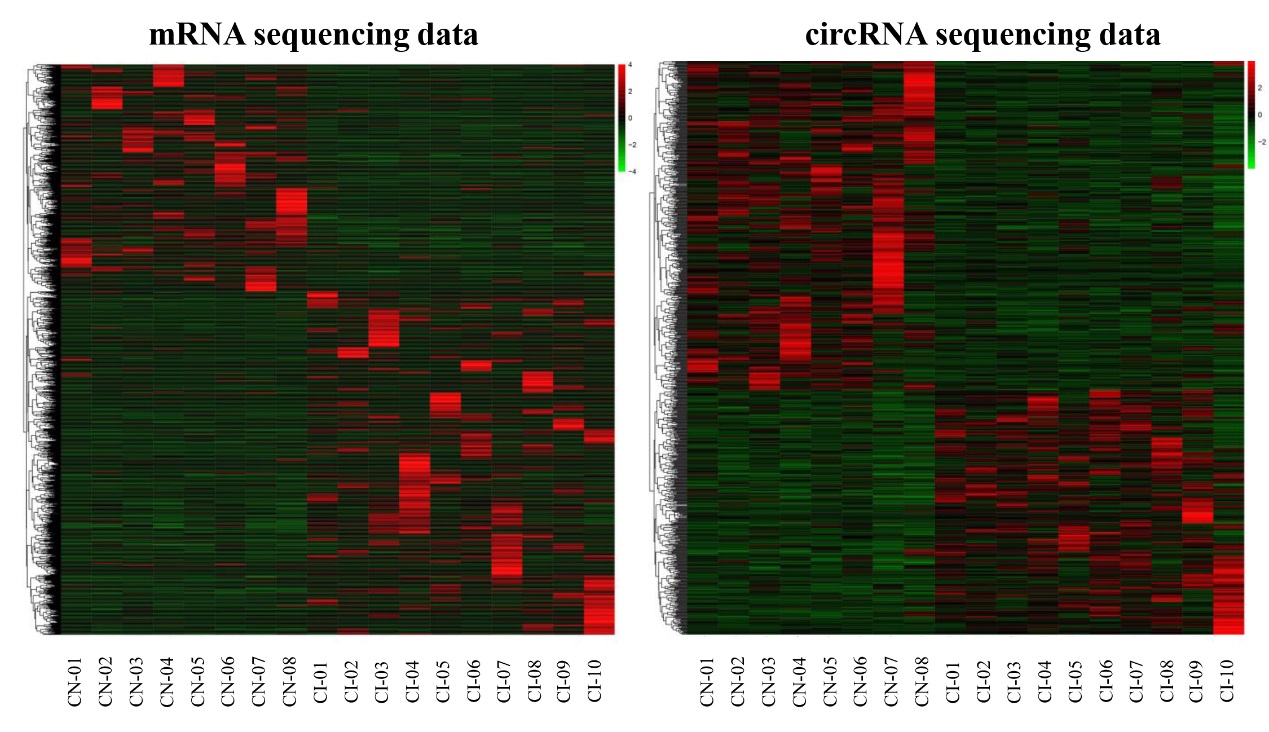


**Supplementary Figure 1. Cluster heat map of mRNAs and circRNAs in cohort 1.**

Left figure shows 1594 differentially expressed mRNAs with more than 2-fold change in expression, and right figure shows 847 differentially expressed circRNAs with more than 2-fold change in expression between 8 CSVD-CN patients and 10 CSVD-CI patients. Red represents high relative expression; green represents low relative expression.


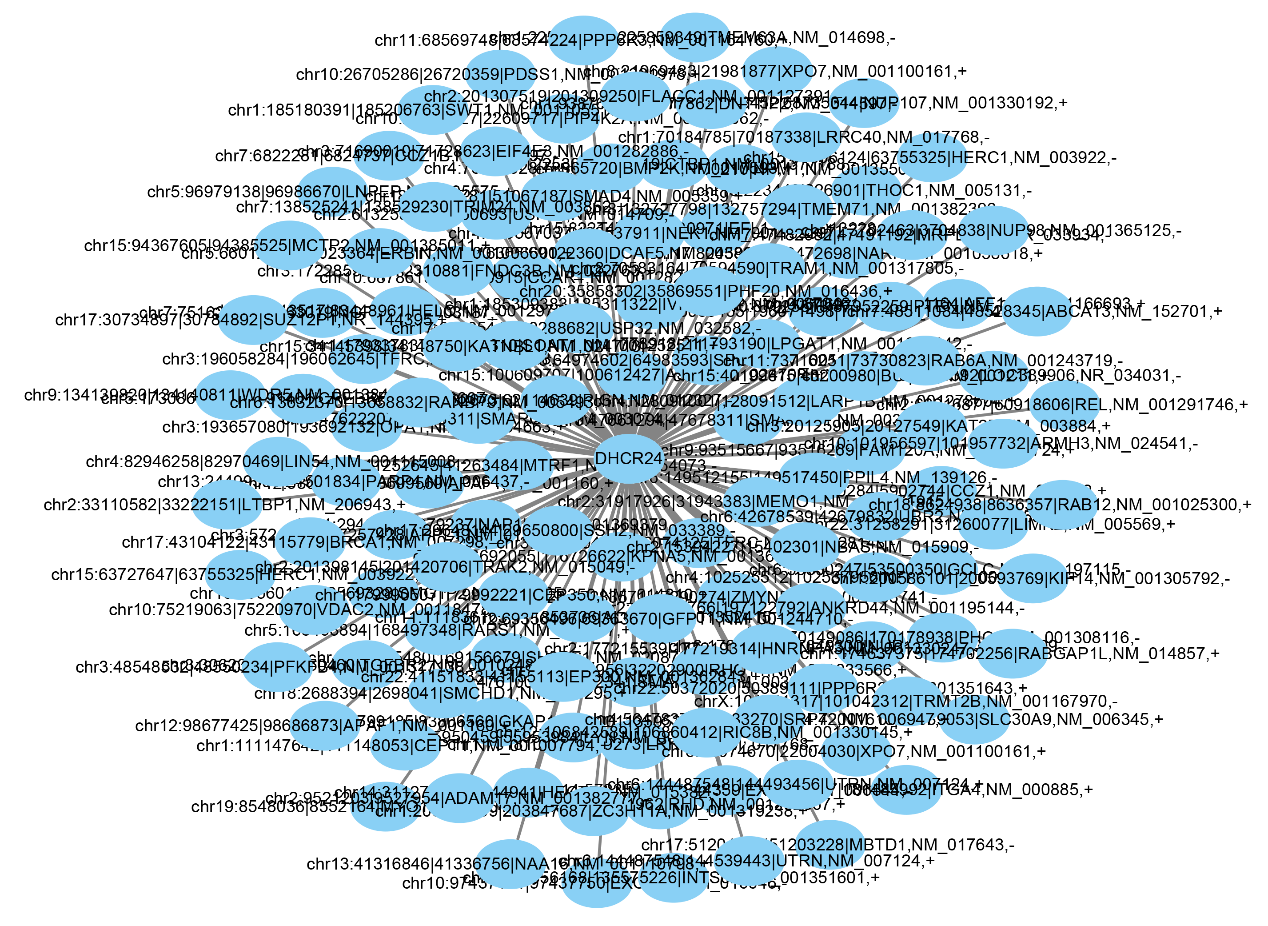


**Supplementary Figure 2. CircRNA-associated DHCR24 networks.**

All networks show a r-value ≥ 0.3 and a p-value < 0.05.


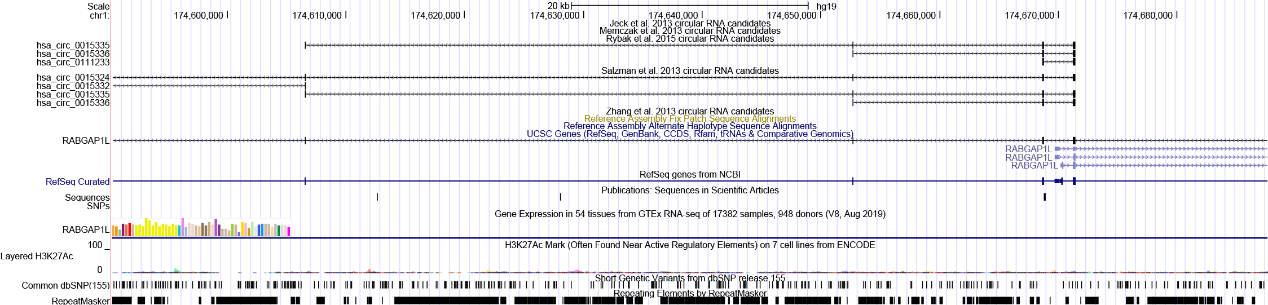


**Supplementary Figure 3. The location of hsa_circ_0015335 in the RABGAP1L gene**


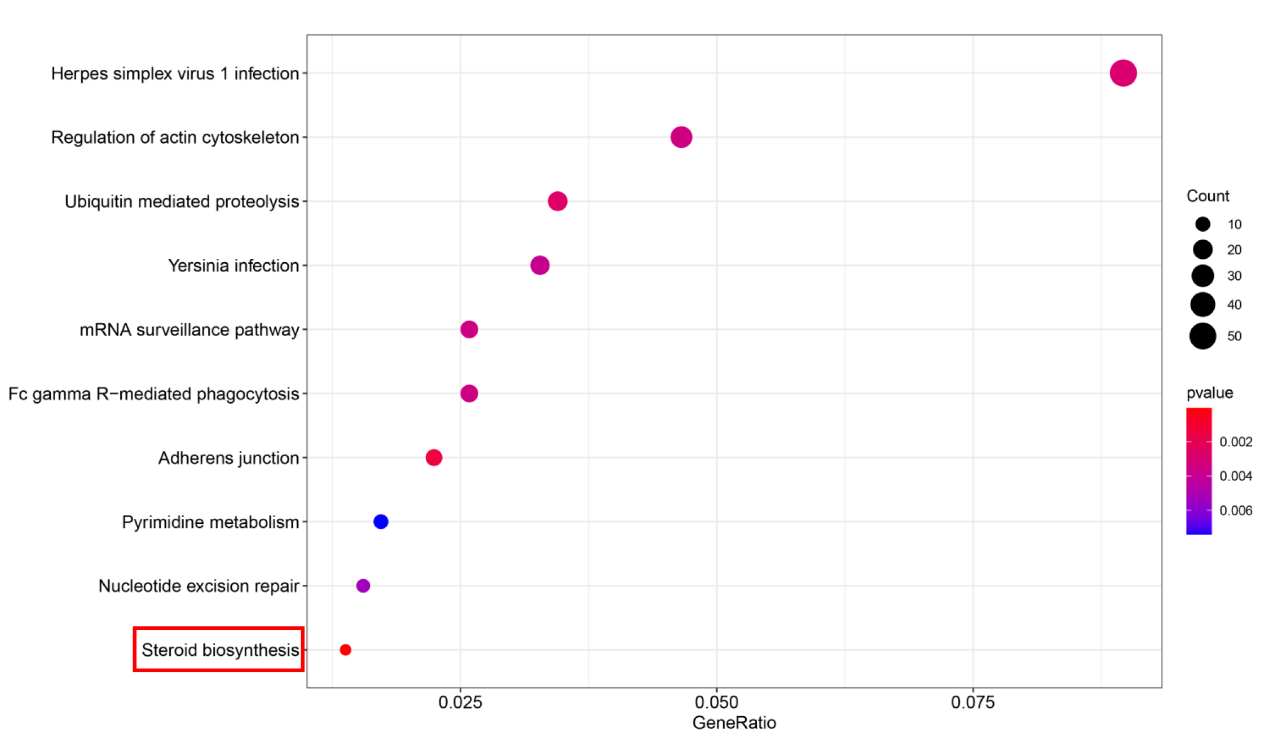


**Supplementary Figure 4. KEGG functional enrichment analysis of mRNA sequencing**

Steroid biosynthesis represents a significantly differential functional mechanism between CSVD-CN and CSVD-CI groups in cohort 1 (adjust p-value = 0.01225.), which is highlighted by the red box.


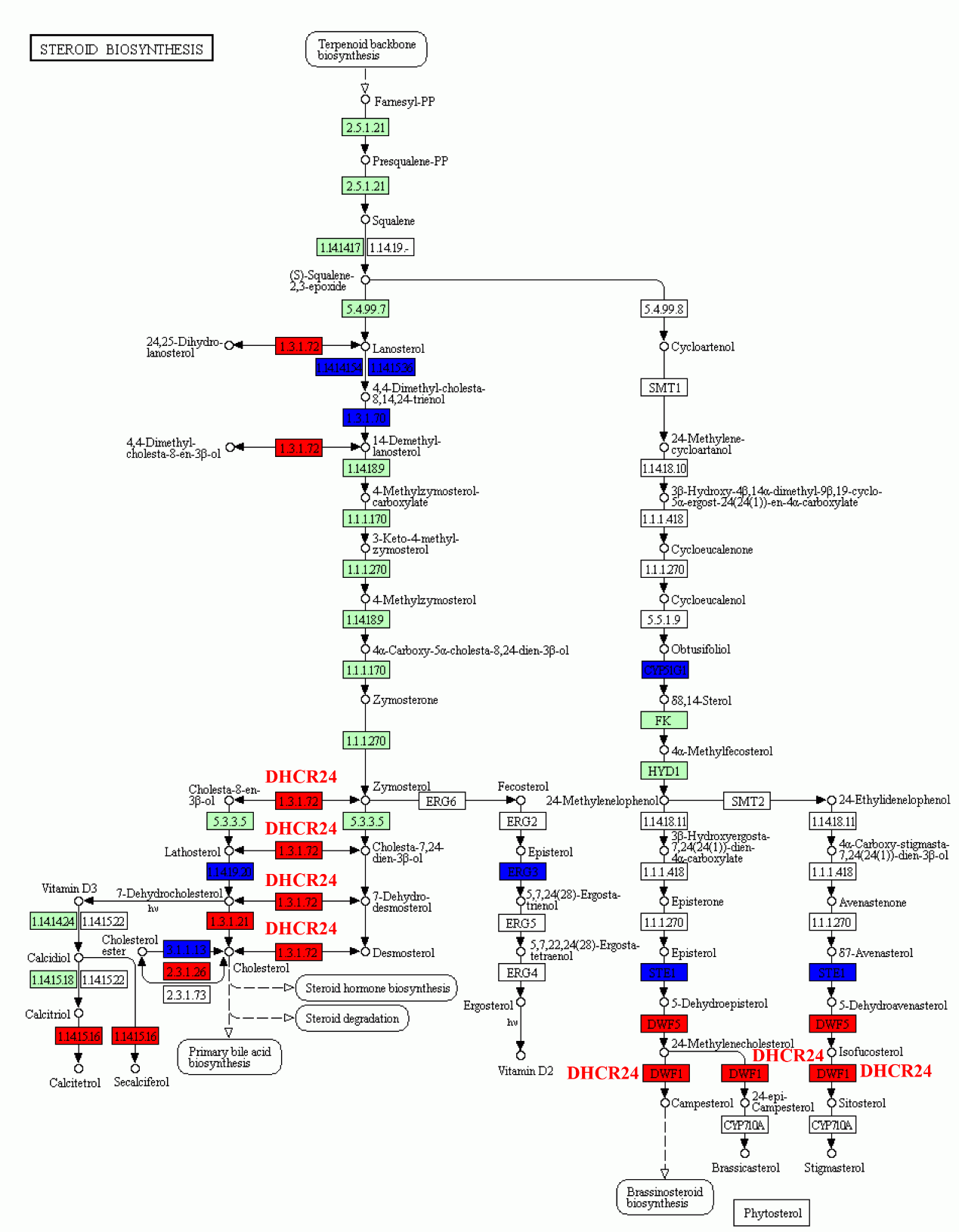


**Supplementary Figure 5. KEGG functional pathway of DHCR24**

KEGG ID is hsa00100, and the link is <https://www.kegg.jp/kegg-bin/show_pathway?hsa00100/6309%09blue/3930%09blue/1595%09blue/3988%09blue/1717%09red/1718%09red/6646%09red/1591%09red>.

# Reference

[1]. Fischl B. FreeSurfer. NeuroImage 2012 62**:** 774-781. <https://doi.org/https://doi.org/10.1016/j.neuroimage.2012.01.021>

[2]. Yousaf T, Dervenoulas G, Politis M. Advances in MRI Methodology. Int Rev Neurobiol 2018 141**:** 31-76. <https://doi.org/10.1016/bs.irn.2018.08.008>

[3]. Kong L, Herold CJ, Zöllner F, Salat DH, Lässer MM*, et al.* Comparison of grey matter volume and thickness for analysing cortical changes in chronic schizophrenia: a matter of surface area, grey/white matter intensity contrast, and curvature. Psychiatry Res 2015 231**:** 176-183. <https://doi.org/10.1016/j.pscychresns.2014.12.004>

[4]. Ségonne F, Dale AM, Busa E, Glessner M, Salat D*, et al.* A hybrid approach to the skull stripping problem in MRI. Neuroimage 2004 22**:** 1060-1075. <https://doi.org/10.1016/j.neuroimage.2004.03.032>

[5]. Fischl B, Dale AM. Measuring the thickness of the human cerebral cortex from magnetic resonance images. Proc Natl Acad Sci U S A 2000 97**:** 11050-11055. <https://doi.org/10.1073/pnas.200033797>
